# Cardiac effects of induction agents in the septic rat heart

**DOI:** 10.1186/cc8038

**Published:** 2009-09-08

**Authors:** York A Zausig, Hendrik Busse, Dirk Lunz, Barbara Sinner, Wolfgang Zink, Bernhard M Graf

**Affiliations:** 1 Department of Anaesthesiology and Critical Care, University of Regensburg, Franz-Josef-Strauss-Allee 11, Regensburg, 93053, Germany

## Abstract

**Introduction:**

The current debate about the side effects of induction agents, e.g. possible adrenal suppression through etomidate, emphasizes the relevance of choosing the correct induction agent in septic patients. However, cardiovascular depression is still the most prominent adverse effect of these agents, and might be especially hazardous in septic patients presenting with a biventricular cardiac dysfunction - or so-called septic cardiomyopathy. Therefore, we tested the dose-response direct cardiac effects of clinically available induction agents in an isolated septic rat heart model.

**Methods:**

A polymicrobial sepsis was induced via cecal ligation and single puncture. Hearts (n = 50) were isolated and randomly assigned to five groups, each receiving etomidate, s(+)-ketamine, midazolam, propofol, or methohexitone at concentrations of 1 × 10^-8 ^to 1 × 10^-4 ^M. Left ventricular pressure, contractility and lusitropy, and coronary flow were measured. Cardiac work, myocardial oxygen delivery, oxygen consumption, and percentage of oxygen extraction were calculated.

**Results:**

All of the induction agents tested showed a dose-dependent depression of cardiac work. Maximal cardiac work dysfunction occurred in the rank order of s(+)-ketamine (-6%) <etomidate (-17%) <methohexitone (-31%) <midazolam (-38%) <propofol (-50%). In addition, propofol showed a maximum decrease in contractility of -38%, a reduction in lusitropy of -44%, and a direct vasodilator effect by increasing coronary flow by +29%.

**Conclusions:**

Overall, this study demonstrates that these tested drugs indeed have differential direct cardiac effects in the isolated septic heart. Propofol showed the most pronounced adverse direct cardiac effects. In contrast, S(+)ketamine showed cardiovascular stability over a wide range of concentrations, and might therefore be a beneficial alternative to etomidate.

## Introduction

Although the ideal induction agent for critically ill patients has not yet been found, there is general agreement that in those patients an induction agent that provides cardiovascular stability upon induction of anesthesia would be first choice. Nevertheless, current guidelines do not recommend one induction agent over another [1,2]. However, there are concerns that non-cardiovascular side effects, such as possible adrenal suppression by etomidate, could compromise critically ill patients and last at least 24 hours [3]. At present the clinical consequences are not clear [4].

However, the most significant adverse effect of induction agents is cardiovascular depression, which has already been well described in healthy animal models and humans after intravenous administration. The degree of negative cardiovascular effects depends on dose and speed of administration and appears to vary greatly among the commonly used drugs [5,6]. In non-septic patients or experimental settings, clinically available induction agents, such as etomidate, propofol, ketamine, methohexitone or midazolam, show dose-dependent effects [5,6]. These effects result from their variable impact on peripheral arteriolar and venous dilation, from direct cardiac depression or both. Surprisingly, direct cardiac effects of induction agents in isolated septic hearts have so far not been systematically evaluated.

The cardiovascular dysfunction in sepsis derives from a reduced systemic vascular resistance typically complicated by decreased cardiac function [1,2]. This cardiac dysfunction - the so-called septic cardiomyopathy - is a major contributor to sepsis-related morbidity and mortality [7,8]. It affects both ventricles in the phases of contraction and relaxation [7-11]. Almost one-fifth of all septic patients with refractory hypotension die because of a low cardiac output deriving from this severe myocardial dysfunction. It is, therefore, the everyday clinical challenge of each intensive care unit physician to sufficiently treat septic patients without further compromising the already reduced function of the septic heart [9]. This mechanical impairment is accompanied by disturbed myocardial metabolism and coronary flow, which influences a balanced myocardial oxygen supply-demand ratio [10].

However, global cardiac mechanical and metabolic effects of these induction agents in septic cardiomyopathy have thus far not been systematically compared in a dose-dependent fashion. There is very little evidence on the direct *in vitro *effects of these agents on cardiac contractile function in sepsis, and the isolated, dose-dependent effects of these induction agents on myocardial excitability, contractility, coronary flow, and oxygen utilization in a septic heart are still unknown. We used the isolated *ex vivo *heart model to study the direct cardiac effects in the absence of confounding neurohormonal, metabolic, or systemic factors.

Therefore, the aim of this study was to directly compare electrical, mechanical, and metabolic effects of etomidate, s(+)-ketamine, midazolam, propofol, and methohexitone at equimolar concentrations, with special emphasis on their impact on cardiac work.

## Materials and methods

Approval from the Institutional Animal Care Committee of the University of Goettingen was obtained before initiation of this study. All experimental procedures conformed with German animal safety regulations. Fifty male Wistar rats (weighing 245 ± 3 g) were injected intraperitoneally with 100 mg/kg ketamine and 2.5 to 5 mg/kg xylazine hydrochloride. A polymicrobial sepsis was induced via cecal ligation and a single puncture as reported previously in detail [12]. After 20 hours of incubation, hearts were isolated and prepared as has been described in recent reports [13]. All hearts were perfused at a perfusion pressure of 55 mmHg with a modified Krebs-Ringer's salt solution, which was filtered in-line (5 μm pore-size filter disk, Sigma-Aldrich^®^, Munich, Germany) and had the following composition: Na^+ ^140 mM; K^+ ^4.5 mM; Mg^2+ ^1.2 mM; Ca^2+ ^2.5 mM; Cl^- ^134 mM; HCO_3_^- ^15.5 mM; H_2_PO_4_^- ^1.2 mM; EDTA 0.05 mM; glucose 11.5 mM; pyruvate 2 mM; mannitol 10 mM; and insulin 5 U/L. Mean aortic inflow pH, partial pressure of carbon dioxide (pCO_2_), and partial pressure of oxygen (PO_2_) were 7.39 ± 0.01, 36 ± 1 mmHg, and 580 ± 25 mmHg, respectively. Perfusate and heart temperature was maintained at 36.9 ± 0.3°C throughout the experiment.

Spontaneous atrial rate, atrio-ventricular conduction time, and systolic left ventricular pressure (LVP) and its derivative were measured as detailed previously [13]. Coronary inflow was measured at constant temperature and under constant pressure of 55 mmHg by a transit-time in-line ultrasound flow meter (Research Flowmeter T106, Transonic Systems, Ithaca, USA). Coronary inflow and outflow (coronary sinus) oxygen tensions (mmHg) were measured discontinuously using a self-calibrating gas analyzer (AVL OMNI 9^®^, Roche Diagnostic, Mannheim, Germany). Oxygen delivery, percent oxygen extraction, and myocardial oxygen consumption were calculated as noted previously [6,13]. Cardiac work ((left ventricular systolic pressure - left ventricular diastolic pressure) × heart rate) was calculated [14]. All measurements were taken during the last minute of each 15-minute experimental period for statistical analysis.

**Figure 1:**
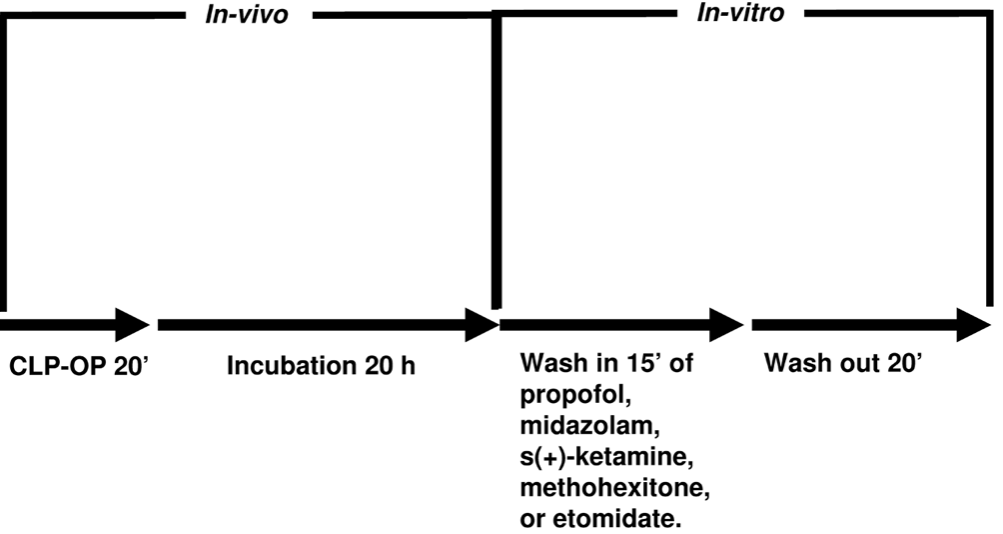
Study protocol. CLP-OP = cecal ligation and puncture operating procedure.

The experimental protocol is shown in Figure 1. After steady state, the hearts were randomly assigned by lottery to five groups (10 hearts each) and received propofol (Disoprivan^®^, AstraZeneca, Wedel, Germany), midazolam (Midazolam-Ratiopharm^®^, Ratiopharm, Ulm, Germany), s(+)-ketamine (S(+)-Ketamine^®^, Pfizer Pharma, Berlin, Germany), methohexitone (Brevimytal^®^, Hikma Pharma, Graefeling, Germany), or etomidate (Etomidat-Lipuro^®^, B. Braun, Melsungen, Germany). Each heart was perfused, in randomized order, at concentrations of 10^-8 ^to 10^-4 ^M with one of these drugs for a period of 15 minutes. There was a 20-minute drug-free washout period. Prior to this study we tested higher concentrations for each of these drugs. However, at concentrations higher than 10^-3 ^M some hearts showed cardiac arrest. Therefore, concentrations of 10^-3 ^M or more were not included in the present study.

The concentrations tested in our study (10^-8 ^to 10^-4 ^M), which are equivalent to 0.002 to 18 μg/mL propofol (molecular weight: 178.3 mM), 0.003 to 33 μg/mL midazolam (325.8 mM), 0.003 to 27 μg/mL s(+)-ketamine (274.2 mM), 0.003 to 26 μg/mL methohexitone (262.3 mM), and 0.002 to 24 μg/mL etomidate (244.3 mM), correspond to approximate therapeutic plasma-free values (corrected for plasma protein binding, in %) of 5.1 to 11 × 10^-7 ^(97 to 98%) propofol, 3.7 to 37 × 10^-9 ^M (94 to 95%) midazolam, 3.2 to 19 × 10^-6 ^M (12 to 30%) s(+)-ketamine, 4.6 to 9.1 × 10^-6 ^M (70 to 73%) methohexitone, and 0.9 to 4.7 × 10^-7 ^M (77 to 94%) etomidate [6,15,16]. However, even higher concentrations up to 10 fold can easily be achieved by bolus injection [17].

### Statistical analysis

All data in the text, tables and figures are displayed as means ± standard error of the mean. Raw data from each functional and metabolic variable were compared by analysis of variance with repeated measures. If F tests were significant, Bonferoni tests were used to compare absolute group means for each variable measured at the same concentration and individual drug concentrations (and washout, WASH) against the initial control (CTRL). *P *< 0.05 was considered to be statistically significant.

## Results

Control values of sham-operated hearts (heart rate 309 ± 4 beats/min, LVP contractility (+dLVP/dt) 3275 ± 84 mmHg/sec, LVP relaxation (-dLVP/dt) 2629 ± 74 mmHg/sec, cardiac work 36036 ± 639 mmHg/beats, and myocardial oxygen supply (DO_2_)/myocardial oxygen consumption (MVO_2_) ratio 1.5 ± 0.0 were statistically different from control values of septic hearts. Sham-operated hearts showed control values of etomidate, s(+)-ketamine, midazolam, propofol, and methohexitone in septic hearts were not statistically different between the groups. After a washout period, each parameter returned to baseline level.

**Figure 2:**
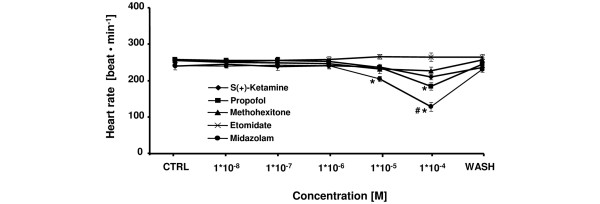
Comparative effects of etomidate, s(+)-ketamine, midazolam, propofol, and methohexitone on heart rate in rat isolated septic hearts. All drugs except etomidate decreased chronotropic effects. For control values, only the first (CTRL) and the washout (WASH) periods are displayed. After the washout period, the heart rate returned to baseline level. *P < 0.05 midazolam (10^-5 ^to 10^-4 ^M) and propofol (10^-4 ^M) versus control; ^#^*P *< 0.05 midazolam versus etomidate, s(+)-ketamine, propofol, and methohexitone. Data are the means ± standard error of the mean.

The comparative effects of etomidate, s(+)-ketamine, midazolam, propofol, and methohexitone on heart rate are shown in Figure 2. No effects on heart rate were observed at 1 × 10^-8 ^to 1 × 10^-6 ^M for any induction agent. At higher concentrations, heart rate was significantly suppressed at 1 × 10^-4 ^M for propofol (maximum decrease: -29 ± 4%) and at 1 × 10^-5 ^to 1 × 10^-4 ^M for midazolam (maximum decrease: -47 ± 5%). Reduction of heart rate by midazolam at 1 × 10^-4 ^M was significantly more pronounced compared with all other tested induction agents. Maximum decreases in heart rate were -12 ± 3% for s(+)-ketamine (1 × 10^-4 ^M) and -11 ± 4% for methohexitone (1 × 10^-4 ^M). Only etomidate showed no chronotropic effect at any tested concentration in this study.

**Figure 3:**
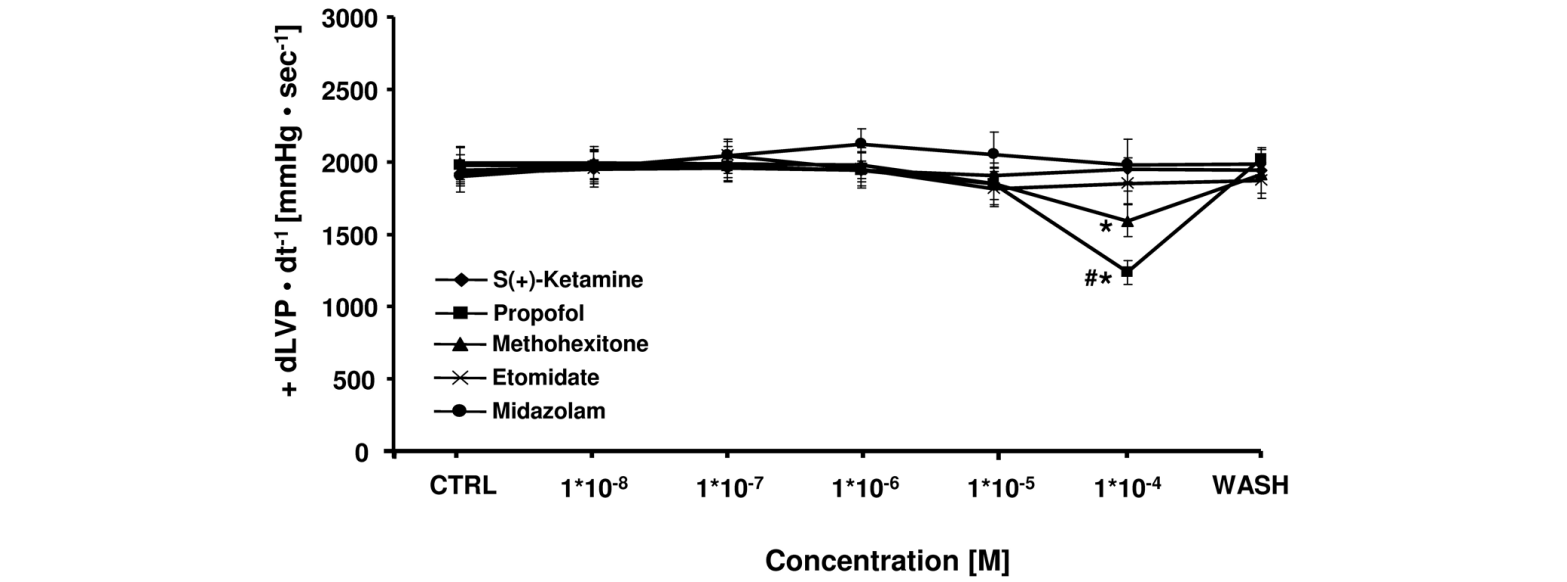
Comparative effects of etomidate, s(+)-ketamine, midazolam, propofol, and methohexitone on left ventricular contractility in rat isolated septic hearts. All drugs except for s(+)ketamine and midazolam decreased contractility. For control values, only the first (CTRL) and the washout (WASH) periods are displayed. After the washout period, left ventricular contractility (+ dLVP/dt^-1^) returned to baseline level. *P < 0.05 for methohexitone and propofol vs. control; ^#^*P *< 0.05 propofol vs. etomidate, s(+)-ketamine, midazolam, and methohexitone. Data are the means ± standard error of the mean.

**Figure 4:**
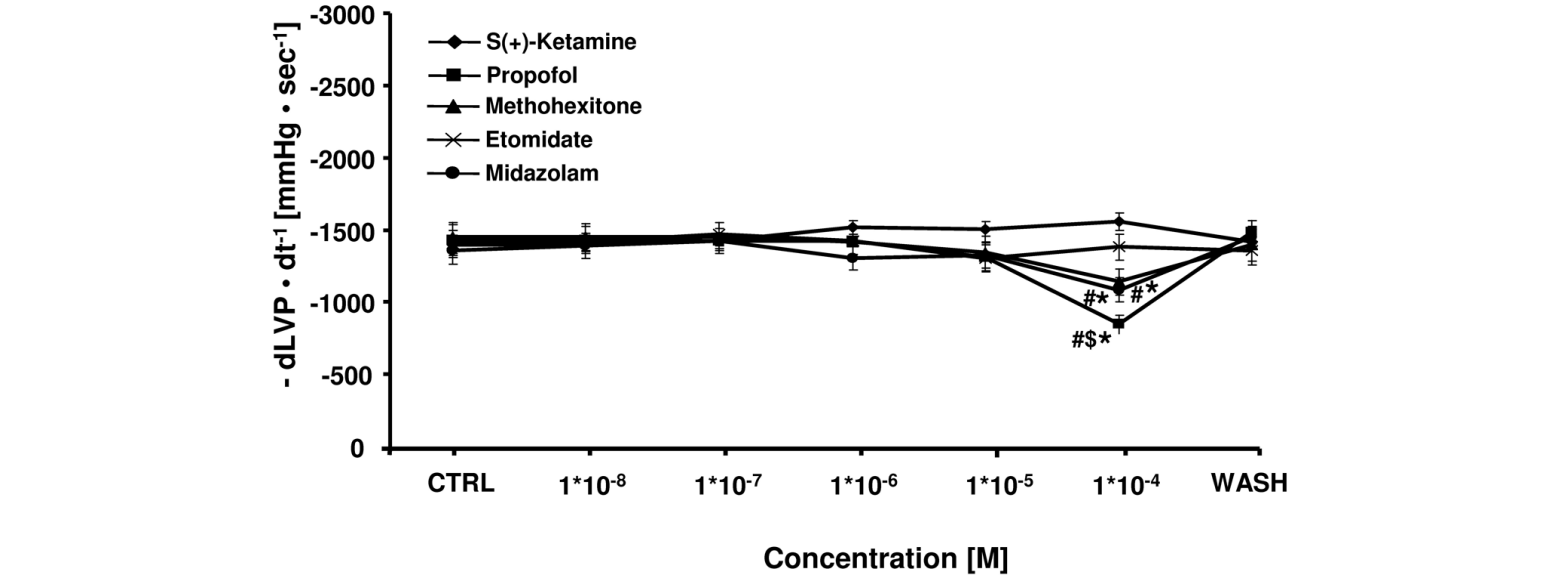
Comparative effects of etomidate, s(+)-ketamine, midazolam, propofol, and methohexitone on left ventricular relaxation in rat isolated septic hearts. All drugs except for s(+)-ketamine decreased lusitropy. For control values, only the first (CTRL) and the washout (WASH) periods are displayed. After the washout period left ventricular relaxation (-dLVP/dt^-1^) returned to baseline level. *P < 0.05 midazolam, propofol, and methohexitone vs. control; ^#^*P *< 0.05 midazolam, propofol, and methohexitone vs. etomidate and s(+)-ketamine; ^$^*P *< 0.05 propofol vs. midazolam and methohexitone. Data are the means ± standard error of the mean.

All tested induction agents showed a dose-dependent decrease in cardiac contractility except for midazolam and s(+)-ketamine (Figure 3). The maximum decrease in +dLVP/dt of -38 ± 5% at 1 × 10^-4 ^M and -19 ± 5% at 1 × 10^-4 ^M was significant for propofol and methohexitone, respectively. The effects of propofol were significantly more pronounced compared with all other agents tested at equimolar concentrations. Other induction agents showed a maximum decrease in contractility of -5 ± 6% for etomidate at 1 × 10^-5 ^M, and a maximum increase in contractility of +7 ± 5% for s(+)-ketamine at 1 × 10^-4 ^M, and +9 ± 6% for midazolam at 1 × 10^-6 ^M. As shown in Figure 4, etomidate, midazolam, methohexitone, and propofol showed negative lusitropic effects with maximal decreases in -dLVP/dt of -7 ± 6% (at 1 × 10^-5 ^M, not significant), -21 ± 5% (at 1 × 10^-4 ^M, significant), -21 ± 6% (at 1 × 10^-4 ^M, significant), and -44 ± 5% (at 1 × 10^-4 ^M, significant), respectively. At 1 × 10^-4 ^M the negative reduction of lusitropy by propofol was significantly different compared with all other tested induction agents. In contrast, at 1 × 10^-4 ^M s(+)-ketamine demonstrated an increase in lusitropy of +14 ± 6%. There was a significant difference compared with propofol and midazolam at equimolar concentration.

**Figure 5:**
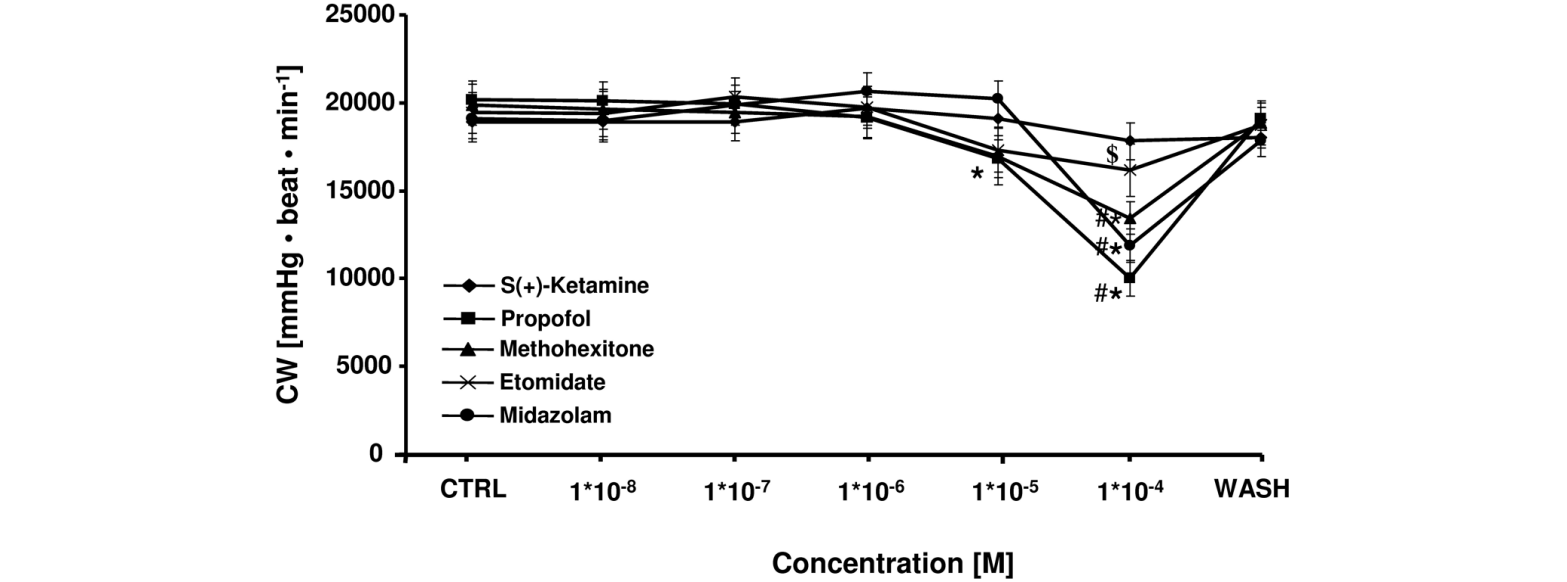
Comparative effects of etomidate, s(+)-ketamine, midazolam, propofol, and methohexitone on cardiac work in rat isolated septic hearts. Each drug decreased cardiac work (CW). For control values, only the first (CTRL) and the washout (WASH) periods are displayed. After washout period CW returned to baseline level. *P < 0.05 midazolam (10^-4 ^M), propofol (10^-5 ^to 10^-4 ^M), and methohexitone (10^-4 ^M) vs. control; ^#^*P *< 0.05 midazolam and propofol vs. etomidate and methohexitone. ^$^*P *< 0.05 s(+)-ketamine vs. propofol, midazolam, and methohexitone. Data are the means ± standard error of the mean.

Cardiac work (Figure 5) - the product of LVP and heart rate - was reduced at 1 × 10^-4 ^M by etomidate (maximum decrease: -17 ± 6%), s(+)-ketamine (-6 ± 6%), midazolam (-38 ± 7%), propofol (-50 ± 6%), and methohexitone (-31 ± 4%) in a dose-dependent fashion. At this concentration, the reduction of cardiac performance was significantly different for propofol, midazolam and methohexitone compared with s(+)-ketamine. Additionally, propofol significantly decreased cardiac work at 1 × 10^-5 ^M by -17 ± 4%.

**Figure 6:**
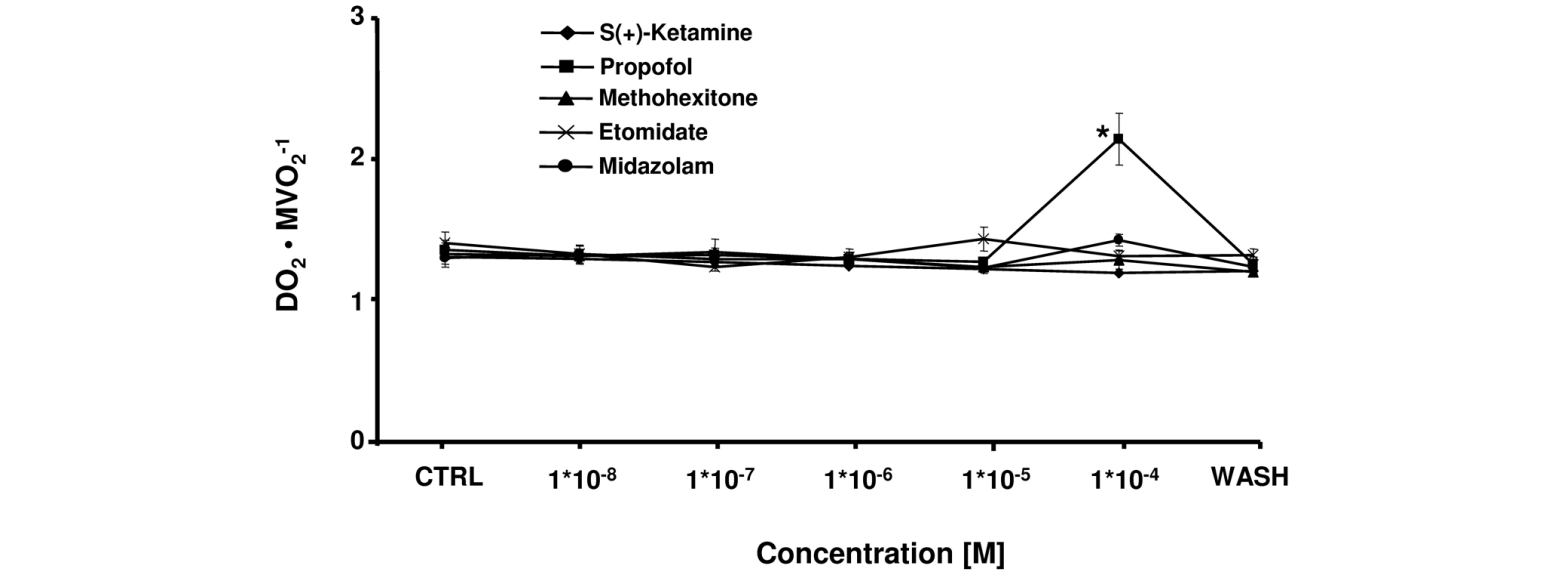
Comparative effects of etomidate, s(+)-ketamine, midazolam, propofol, and methohexitone on myocardial oxygen supply/myocardial oxygen consumption in rat isolated septic hearts. All drugs decreased myocardial oxygen supply/myocardial oxygen consumption (DO_2_/MVO_2_^-1^) ratio. For control values, only the first (CTRL) and the washout (WASH) periods are displayed. After the washout period DO_2_/MVO_2_^-1 ^returned to baseline level. *P < 0.05 propofol vs. control, etomidate, s(+)-ketamine, midazolam, and methohexitone. Data are the means ± standard error of the mean.

Etomidate, s(+)-ketamine, midazolam, and methohexitone showed no direct effects on coronary flow, myocardial oxygen supply and demand (all not shown). Therefore, DO_2_/MVO_2 _ratio (Figure 6) was not affected by these agents. These effects were similar for propofol at 1 × 10^-8 ^to 1 × 10^-5 ^M. However, at 1 × 10^-4 ^M propofol significantly increased coronary flow of +29 ± 4%. Additionally, there was a considerable cardiac-work induced decrease in MVO_2 _and oxygen extraction, accompanied by a coronary flow dependent rise of DO_2 _leading to increase of DO_2_/MVO_2 _ratio (Figure 6) of +58 ± 4%. This was significantly different compared with etomidate, s(+)-ketamine, midazolam, and methohexitone at equimolar concentration.

## Discussion

The study was designed to compare the direct effects of five commonly used intravenous induction agents by analyzing cardiac responses at equimolar concentrations in septic hearts. The tested drugs demonstrate differential direct effects on electrical properties, myocardial function, andoxygen supply-to-demand ratio. Propofol showed the most pronounced adverse direct cardiac effects, whereas s(+)ketamine was most beneficial, as it showed cardiac functionality over a wide range of concentration.

There are concerns regarding the application of etomidate in critically ill patients, especially in septic patients due to possible adrenal suppression [18]. The incidence of this adrenal suppression in sepsis ranges from 9 to 67%, and cortisol response to corticotrophin is more frequently impaired after administration of etomidate as compared with alternative induction agents [19]. However, in septic patients, cardiovascular instability is the main focus of clinicians because it is the major cause of morbidity and mortality in sepsis. The presence of cardiac dysfunction - demonstrated as septic cardiomyopathy - additionally decreases survival rate in septic patients [10]. Therefore, an induction agent that provides cardiovascular stability such as etomidate is frequently used in healthy subjects as it is intended to show minimal cardiovascular effects [4,5]. In the present study, we show that etomidate is safe with regard to cardiac function at concentrations of 10^-8 ^to 10^-5 ^M in septic hearts. However, at higher concentrations it markedly depresses cardiac work. These concentrations can easily be achieved either by bolus administration or by long-term infusion in patients with severe sepsis or septic shock, especially with multiple-organ failure accompanied by a decreased hepatic and renal metabolism [20]. Therefore, these effects must be kept in mind, especially because other studies underline that a higher induction dose of etomidate is also associated with a decrease in systolic arterial blood pressure in animal models and patients with advanced age and heart disease [21].

In contrast, s(+)ketamine showed cardiac functionality over a wide range of concentrations. S(+)ketamine is an optical isomer of ketamine and exhibits stereoselective bindings to different receptors, accounting for its three to four times higher anesthetic potency compared with the R(-)-isomer [16,22]. The racemic ketamine and both ketamine stereoisomers show negative chronotrope, dromotrope, and inotrope effects in the isolated healthy heart [22]. In septic hearts, s(+)ketamine has no significant negative effect on LVP, contractility or lusitropy. This discrepancy might be explained by the fact that both the R(-)-isomer and racemic ketamine in general show significantly more cardio-depressant effects as compared with the S(+)-isomer [16,22]. The mechanism behind this is a stereoselective suppression of the trans-sarcolemmal Ca^2+ ^current (ICa^2+^), which play an important role in the force of contraction and spontaneous firing of sinoatrial node cells, as demonstrated in electrophysiological experiments [23].

Midazolam and methohexitone, together with propofol, showed the most adverse effects on cardiac stability. Propofol, midazolam and methohexitone decreased cardiac work in a dose-dependent fashion. At very similar concentrations, Stowe and colleagues showed a decrease in contractility in guinea pig hearts from midazolam, propofol, and thiopental [6]. However, the degree of contractility reduction was more pronounced in healthy hearts as compared with septic ones. These surprisingly different results might be model or protocol dependent. Otherwise, as the mechanisms of the cardiac depressant effects of these induction agents is likely to involve attenuation of trans-sarcolemmal Ca^2+ ^flux [6], the dysfunction of sarcoplasmic reticulum Ca^2+ ^handling or altered calcium transient properties described in septic hearts might be attributable to these differing results [24,25]. The most striking finding on coronary flow was a direct vasodilating effect by propofol at 1 × 10^-4 ^M. This effect suggests that coronary autoregulation was inhibited at this concentration, and propofol may cause a substantial coronary vasodilation when used as an anesthetic induction agent [6,13]. In contrast, no other tested induction agent showed a direct vasodilating effect at any concentration. However, care has to be taken because depression of heart function is not always an expression of hazardous effects. For example, vasodilatation of the coronary arteries induced by propofol might have led to improving myocardial blood and oxygen supply as shown in Figure 6. Additionally, the slow down of the heart rate by midazolam might reduce myocardial energy demands, and may additionally improve diastolic filling of the heart.

We recognise the limitations of this study. Although the applied sepsis method has the advantages of inducing a 'natural' course of infection, it has limitations with regard to noteworthy outcome variability [26,27]. In contrast, other sepsis models, such as the bolus injection-type method, offer a simple and highly standardized method. However, failure of transmission of therapeutic results from bolus shock experiments into clinical use has emphasized that these models do not reflect all aspects of the sepsis syndrome [26]. In contrast, the cecal ligation and single puncture method is generally recognized as closely mimicing human disease by activating pro- and anti-inflammatory pathways. Another limitation of this study is that in addition to cardiac depression, induction agents also induce a systemic vascular dilatation that leads to hypotension. This is associated with an increased risk of death in critically ill patients [28]. However, the diagnosis of hypotension is easy, whereas the diagnosis of septic cardiomyopathy is more sophisticated and requires a more complex analysis. Therefore, at the moment of induction, this diagnosis may not be available, and septic patients would be at an increased risk in terms of choosing the wrong induction agent. On this account, we used an *ex vivo *approach and isolated hearts and focused on the direct cardiac effects of the applied induction agents. The advantages of this method are to measure mechanical and metabolic properties in the absence of the confounding effects of other organs, systemic circulation, and a host of peripheral complications such as circulating neurohormonal factors [29]. One potential limitation of an isolated heart preparation study is the possible influence of a force-frequency relationship. Although there are significant changes in heart rate for midazolam, which are not accompanied by a significant change in +dLVP/dt (Figure 3) at 10^-5 ^M, the possible influence of a force-frequency relationship has to be kept in mind when interpretating the presented results.

## Conclusions

In conclusion, this study showed that the tested drugs - etomidate, s(+)-ketamine, midazolam, propofol, and methohexitone - indeed have differential direct cardiac effects, even in the isolated septic heart. Propofol showed the most pronounced adverse direct cardiac effects, while S(+)ketamine demonstrated cardiac stability over a wide range of concentrations. Thus, if our data can be extrapolated to apply to humans, it seems that there are alternatives to etomidate such as s(+)ketamine, which demonstrates similar cardiac stability, but with less non-cardiovascular side effects affecting the outcome of septic patients.

## Key messages

• Induction agents show differential direct cardiac effects in septic cardiomyopathy.

• propofol show most pronounced adverse effects.

• S(+)ketamine demonstrates cardiac stability over a wide range of concentrations.

## Abbreviations

+dLVP/dt: left ventricular contractility; -dLVP/dt: left ventricular relaxation; DO_2_: myocardial oxygen supply; LVP: Left ventricular pressure; MVO_2_: myocardial oxygen consumption; pCO_2_: partial pressure of carbon dioxide; PO_2_: and partial pressure of oxygen.

## Competing interests

On behalf of my co-authors I attest that the work has not been funded by any source(s) other than described in the statement. No author or participant has any financial interest in the subject matter, materials or equipment discussed or in competing materials. The laboratory in which the research was performed has not been funded by, or has any participant in the planning, conduct, or reporting of the research been funded by or have financial interests in any source with a real or potential interest in the subject matter, materials, equipment or devices discussed or in any competing product or subject. And the laboratory in which the work was performed or any of the authors or participants have not been funded by any foundation or other non-governmental source that has received funding from any organization with a real or potential interest in the subject matter, materials, equipment or devices discussed, or in any competing product or subject.

## Authors' contributions

YZ and BG originated the idea and performed preliminary experiments. HB continued to perform the experiments. BS coordinated to the laboratory support. YZ and WZ were responsible for writing the paper. DL, BS and BG supported the editing of the manuscript and added important comments to the paper. All authors read and approved the final manuscript.

## Authors' information

The data was presented in part at the 3rd International Congress of the German Sepsis Society in Weimar from 5 to 8 September, 2007.
